# Pathological Rupture of the Spleen in Uncomplicated Myeloma

**DOI:** 10.4137/ccrep.s725

**Published:** 2008-04-22

**Authors:** Vincent Ho, Andrew Shearer, Mark Jagusch

**Affiliations:** 1 Clinical Senior Lecturer, School of Medicine, James Cook University, Cairns, Queensland, Australia.; 2 Haematologist, Department of Medicine, Cairns Base Hospital, Cairns, Queensland, Australia.; 3 Head Department of Pathology, Cairns Base Hospital, Cairns, Queensland, Australia.

**Keywords:** spleen, rupture, myeloma

## Abstract

Pathological rupture of the spleen in uncomplicated myeloma is extremely rare. We present a case of a splenic rupture which occurred in a 52 year old woman with uncomplicated multiple myeloma. The patient required an urgent splenectomy and had an uneventful recovery. Pathophysiological mechanisms leading to splenic rupture are discussed. Plasma cell leukaemias have been previously documented to present with splenic rupture. A subgroup of aggressive multiple myelomas such as in our case may have a similar tendency for splenic rupture.

A 52 year old woman was diagnosed with multiple myeloma ISS Stage III in 2006. Her past medical history was significant for diet-controlled diabetes mellitus and hypertension. She had a background of a few months of right hip pain and a pathological femoral fracture had been noted on plain films. A right total hip replacement was performed and a bone specimen revealed infiltration of cells exhibiting features suggestive of plasmacytoma or occult myeloma. Subsequent protein electrophoresis revealed the presence of a lambda protein band of approximately 1 g/l in the gamma region. Her albumin and corrected calcium levels were normal at 40 g/l and 2.36 mmol/l respectively. Her β-2 microglobulin level was recorded as 6.8 mg/l.

A bone marrow biopsy performed revealed 85%–90% marrow infiltration with plasma cells, markedly reduced erythropoiesis and moderately reduced myeloid numbers. She was discharged and plans made for outpatient chemotherapy.

One week later she presented to hospital with acute left pleuritic pain. There was notable tenderness over the lower left ribs anteriorly and back. Cardiovascular, respiratory and neurological systems were normal. Her abdomen was soft to palpation, non-tender and there were no masses detectable. Vital sign recordings, chest X-ray and ECG were normal.

FBC showed a Hb of 99 g/l, WBC 9.0 × 10^9^/l and platelet 136 × 10^9^/l. Blood film revealed numerous atypical plasma cells representing 7% of cells on peripheral blood. Electrolyte profile was unremarkable apart from a mild hyperphosphatemia PO4^3−^ 2.00 mmol/l. Coagulation screen showed INR 1.2, APTT 32 and fibrinogen 3.1 g/l.

An MRI of the spine did not reveal any significant abnormality. A consideration of pulmonary emboli was made as the cause of the pleuritic chest pain and a CT pulmonary angiogram ordered on the 3rd day of admission. She was anticoagulated with subcutaneous enoxaparin pending the result of the investigation. The CT pulmonary angiogram did not reveal pulmonary emboli but a markedly enlarged spleen was noted with a wedge-shaped low density area located posteriorly in the spleen and a small adjacent hyperdense area. The radiological finding was thought to be consistent with a splenic infarct and associated haemorrhagic transformation. See Figure 1.

Subcutaneous enoxaparin was discontinued and the putative splenic infarction was managed conservatively with analgesia.

The next day the patient developed pain in the lower chest radiating to the back and left shoulder, became nauseous, dyspnoeic and diaphoretic. On abdominal examination she had marked tenderness in the left quadrant with a distended abdomen, and was noted to be hypotensive with a blood pressure of 90/50, hypoxic with a saturation of 88% room air and tachycardic at a pulse rate of 120/minute. Peripheral pulses were thready and her skin was mottled. ECG revealed new T-wave inversion in lead 3. Her haemoglobin had fallen to 32 mg/dl from 110 mg/dl the day previous and platelet count fell to 21×10^9^/l. She had been oliguric overnight and her creatinine was recorded at 146 micromol/litre.

A diagnosis of shock secondary to splenic rupture was made and an urgent laparotomy arranged for splenectomy. At laparotomy an extensive tear of the superior pole of the spleen was visualised. 2300 mls of blood in the abdominal cavity was evacuated. Intraoperatively she required 2 litres of crystalloid, 4 units of fresh frozen plasma, 4 units of packed cells and 4 units of platelets. She was admitted to the intensive care unit post-operatively where her condition remained stable. Her recovery was uneventful.

The spleen weighed 712 grams and measured 185 × 130 × 60 mm. On macroscopic assessment at one pole of the spleen there was an extensive area of irregular disruption of the surface involving the lateral and posterior aspects extending to involve the pole itself. On longitudinal sectioning of the spleen there was haemorrhagic disruption and induration of the tissues within the region of the spleen that was underlying the areas of surface rupture and the area involved was approximately 110 × 60 mm. Hilar blood vessels were free of thromboemboli. The remainder of the spleen had a very reddish appearance with loss of the normal follicular architecture. There were no features of external trauma. See Figure 2.

Microscopically considerable interstitial haemorrhage within tissues was visualised and while the major component of this appeared to be recent in origin there were a few foci suggestive of granulation tissue formation.

Morphology of extramedullary haematopoiesis was noted. See Figure 3. Scattered plasma cells were seen about vascular channels and suggest focal infiltration by myeloma.

Features typical of infarction or amyloidosis were not identified.

## Discussion

Spontaneous splenic rupture is an uncommon and potentially fatal clinical entity, well-described in various haematological disorders (Giagounidis et al. 1996).

The risk of splenic rupture in haematological malignancies is known to correlate with the size of the spleen and ruptured spleens are generally moderately to severely enlarged (Giagounidis et al. 1996; Smart et al. 2002). Extramedullary haematopoiesis defined as the appearance of haematopoietic elements outside of the bone marrow and often occurs in tissues such as the liver and spleen (Bain and Gupta, 2003). Extramedullary haematopoiesis is a known cause of splenomegaly.

Spontaneous splenic rupture where extramedullary hematopoiesis was thought to be contributory is considered unusual in multiple myeloma, and has been reported in only 3 cases in the literature (Stephens and Hudson, 1969; Polliack and Hershko, 1972; Okazaki et al. 1986). Another case of spontaneous splenic haematoma has been documented to have occurred in a patient receiving pegfilgrastim (Hatzimichael et al. 2006). Pegfilgrastim is a synthetic long-acting colony-stimulating factor that increases the neutrophil count in patients receiving chemotherapy. The putative mechanism for haematoma from pegfilgrastim was thought to be extramedullary haematopoiesis resulting in splenomegaly thus increasing the tendency to rupture (Litam et al. 1993).

There is only one previously reported case of spontaneous splenic rupture attributable to uncomplicated myeloma (Sherwood et al. 1996). In that case the splenic rupture was hypothesised as attributable to the tumour pressure effect of multiple plasmacytomas on the splenic capsule although this was not definitively established.

It is well established that amyloidosis increases the risk of splenic rupture. This is as a result of amyloid deposition leading to capsular distension and increased vascular fragility (Khan et al. 2003). The mere presence of plasma cells within vessels itself however is not known to increase vascular fragility.

Hynes and colleagues suggested three possible modalities of splenic ruptures in patients with leukaemia: mechanical infiltration of the spleen, splenic infarction and defects in blood coagulation (Hynes et al. 1964).

The first modality appears to be relevant to the plasma cell dyscrasias particularly with the plasma cell leukaemias, where collections of myeloma cells are often seen infiltrating vessels walls right up to the endothelium (Rogers and Shah, 1980). Capsular invasion with plasma cells have been seen in such cases and thereby could substantially weaken the capsular wall at the time of rupture. While not meeting the definition of a plasma cell leukaemia the aggressive behaviour of our patient’s myeloma with extensive infiltration of marrow, high β-2 microglobulin, marked splenomegaly and invasion of splenic vascular channels may account for a similar presentation.

In our case it is unlikely in the absence of infarction at pathological examination that thromboembolism with subsequent infarction and rupture is a causative mechanism.

Defects in coagulation would compound the haemorrhaging associated with capsular invasion and rupture. Our patient received one dose of subcutaneous enoxaparin as empirical treatment for suspected pulmonary venothromboembolism, which would exacerbate any pre-existent bleeding. The absence of bleeding sites other than the spleen at laparotomy however provides evidence against coagulation abnormalities being the predominant cause of splenic rupture.

The hyperdense lesion seen on initial CT scan was attributed to haemorrhagic transformation adjacent to a splenic infarct rather than haemorrhage in the splenic parenchyma. Spontaneous splenic rupture is a far less common radiological finding than splenic infarction (Gorg et al. 2003) and it was not until the ultrasound finding of free intraabdominal fluid that the diagnosis of splenic rupture was made. Abdominal ultrasound has 98%–100% sensitivity for the detection of free intra-abdominal fluid and can be useful for guiding treatment decisions in splenic rupture (Goletti et al. 1994).

Due to the presence of a few foci of granulation tissue a pre-existing splenic injury could not be ruled out but there was no documented history of pre-existing splenic trauma.

Splenic rupture in association with uncomplicated multiple myeloma has been reported to our knowledge only once before and appears to be a very rare complication of a not uncommon haematological malignancy. We hypothesise that occasional forms of multiple myeloma can behave aggressively to resemble features of plasma cell leukaemias, with marked splenomegaly, extramedullary haematopoiesis and infiltration of splenic vessels all contributing to an increased likelihood of splenic rupture.

## Figures and Tables

**Figure 1:**
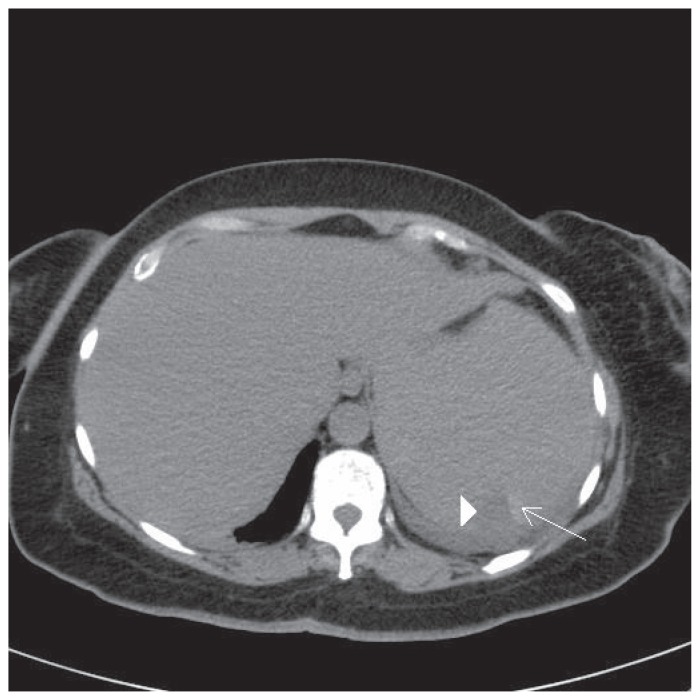
There is a wedge shaped low density area in the posterior spleen (arrowhead) and a hyperdense adjacent area thought to be associated haemorrhagic transformation (long arrow).

**Figure 2:**
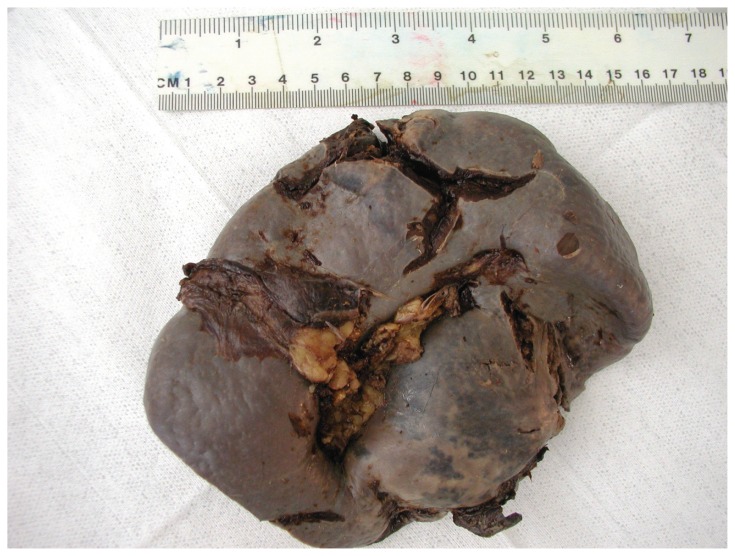
The external appearance of spleen reveals a large tear of the superior pole that extends laterally.

**Figure 3:**
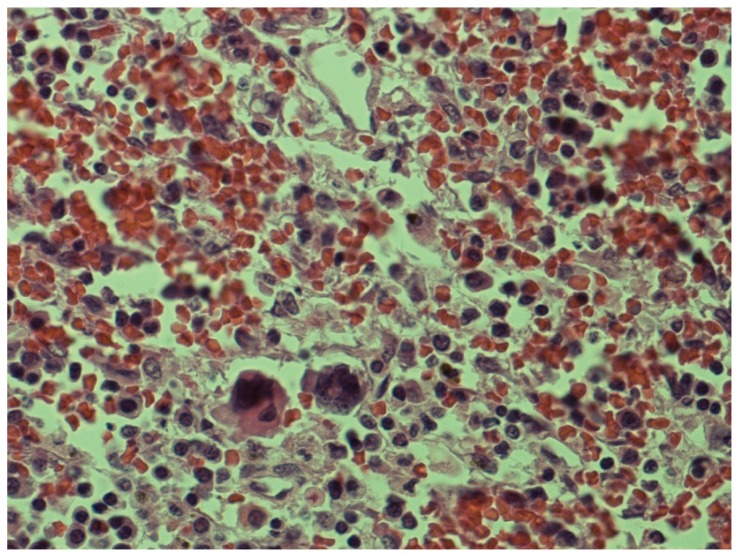
In this photo extramedullary haematopoiesis is evident with a few megakaryocytes seen in the lower left field. There is an abundance of plasma cells seen within the splenic parenchyma.
